# Association Between the Cancer and Aging Research Group Score and Chemotherapy Toxicity in Older Portuguese Patients: A Retrospective Single-Center Study

**DOI:** 10.7759/cureus.104048

**Published:** 2026-02-22

**Authors:** Margarida Lagarto, Tiago Barroso, Patricia Cavaco, Tiago Pina Cabral, Filipa Ferreira, Ana Martins

**Affiliations:** 1 Medical Oncology, Unidade Local de Saúde Lisboa Ocidental, Lisbon, PRT; 2 Medical Oncology, Centro Hospitalar Universitário Lisboa Norte, Lisbon, PRT; 3 Pharmacology, Unidade Local de Saúde Lisboa Ocidental, Lisbon, PRT

**Keywords:** carg score, chemotherapy-related toxicity, geriatric-oncology, older adults, real-world data, risk prediction

## Abstract

Background

Older adults are more likely to be affected by cancer and are at higher risk of chemotherapy‑related toxicity. The Cancer and Aging Research Group (CARG) score was developed to estimate the risk of severe toxicity in older patients receiving chemotherapy, but its performance varies across settings and populations.

Objective

The objective of this study is to evaluate the correlation between the CARG risk score and chemotherapy‑related toxicity in older adults with solid tumors treated at a Portuguese Oncology Center.

Methods

We conducted a retrospective observational study of patients aged ≥65 years with solid malignancies who received chemotherapy between January and December 2023 at our center. Patients treated with immunotherapy or targeted therapies were excluded. Baseline clinical and geriatric variables were collected from electronic medical records. Toxicities were graded using CTCAE v5.0; the maximum toxicity grade per patient was recorded. Spearman’s rank correlation assessed the association between the CARG score and maximum toxicity grade.

Results

A total of 283 patients were included (median age 73 years (range 65-93); 56.2% (n = 159) male; ECOG PS 0 in 65.4% (n = 185)). The median BMI was 25.2 kg/m²; median SCr 0.86 mg/dL; median SHb 12.4 g/dL. Overall, 84.1% (n = 238) experienced at least one chemotherapy‑related adverse event: grade 2 in 49.1% (n = 139) and grade 3-4 in 32.9% (n = 93). Fatigue (n = 88, 31.1%) and neutropenia (n = 80, 28.3%) were the most frequent toxicities. The median CARG score was 6 (range 2-18); 21.9% (n = 62) were low‑risk, 59.4% (n = 168) intermediate‑risk, and 18.7% (n = 53) high‑risk. The incidence of grade 3-4 toxicity increased across CARG categories: low 22.6% (n = 14), intermediate 41.2% (n = 69), and high 79.2% (n = 42). The CARG score correlated significantly with maximum toxicity grade (Spearman R = 0.27; p = 2.8 × 10⁻⁶).

Conclusions

In this real‑world Portuguese cohort of older adults with solid tumors, the CARG score was significantly associated with chemotherapy‑related toxicity, with a stepwise increase in severe events across risk categories. Although the correlation strength was modest, these findings support the use of CARG as a complementary tool to anticipate toxicity and guide shared decision‑making. Prospective studies incorporating modern systemic therapies and capturing the impact of low‑grade toxicities are warranted.

## Introduction

Older age is associated with an increased risk of developing cancer. Individuals 65 years or older have an estimated 11-fold increase in cancer incidence and a 16-fold increase in cancer mortality when compared to younger adults [1]. In Europe, people aged 65 and over account for nearly 60% of all cancer diagnoses, and this proportion is expected to rise as the population continues to age [2,3].

This growing incidence poses a challenge for oncologists, as older adults are a highly heterogeneous group and remain under-represented in clinical trials. A systematic review by Sedrak et al. showed that patients aged 75 years or older represent less than 10% of participants enrolled in National Cancer Institute Cooperative Group Clinical Trials [4]. Moreover, those who are included often do not reflect real-world geriatric patients, as many are excluded due to performance status, comorbidities, or abnormal laboratory parameters [4-6]. 

Existing evidence suggests that older adults have similar chemotherapy benefits to younger individuals; nevertheless, they tend to experience more toxicity [1]. A meta-analysis of five phase III chemotherapy trials reported that 35% of older patients experienced a grade 3 to 4 adverse event (AE) compared with only 26% of younger patients [7]. Overall, approximately 30% to 50% of older adults with solid tumors experience a grade 3 to 5 chemotherapy-related toxicity [8].

Given these challenges, a Comprehensive Geriatric Assessment (CGA) is recommended for older patients with cancer to individualize treatment strategies based on their functional status and expected treatment tolerance. Evidence shows that CGA influences treatment decision making in 20% to 50% of cases and that CGA-based interventions increase the likelihood of completing full-dose chemotherapy, with these patients experiencing fewer dose reductions and fewer treatment-related AEs [9-12].

However, CGA implementation requires time and resources that are not consistently available across institutions, at least in a timely manner. As a result, clinicians often rely on chronological age or Eastern Cooperative Oncology Group Performance Status (ECOG PS), despite their limited ability to predict frailty or chemotherapy toxicity [5,6,13].

The Cancer and Aging Research Group (CARG) score was developed by Hurria et al. as a chemotherapy toxicity prediction tool for patients aged 65 years or older with solid tumors. It incorporates age, tumor type, treatment intensity, selected laboratory measures, and five key geriatric assessment domains to categorize patients into three groups, according to the risk of developing grade 3-5 toxicity: low risk (0 to 5 points, 30%), intermediate risk (6 to 9 points, 52%) and high risk (10 to 19 points, 83%) [1]. This score has been internally validated, and several studies have evaluated its external validity across different populations, tumor types, and treatment lines. Overall, the results were contradictory [2,8,10,13-16].

We conducted a retrospective analysis to assess whether the CARG score correlates with treatment-related toxicity in older adults with solid tumors receiving systemic therapy at a Portuguese Oncology Center.

## Materials and methods

We undertook a retrospective observational analysis using archival data from the oncology electronic medical records of our Oncology Department between January and December 2023. All patients aged 65 years or older who had a diagnosis of a solid malignancy and received chemotherapy during this period were included. Patients receiving immunotherapy or targeted therapies were excluded.

Baseline characteristics, including gender, age, body mass index (BMI), performance status (measured by ECOG), geriatric variables included in the CARG score, serum creatinine (SCr), serum hemoglobin (SHb), tumor type, treatment dosage (standard vs. reduced) and regimen (mono- vs. polychemotherapy), were collected from electronic medical records. The BMI was calculated using the formula: weight (Kg)/ [height (m)]^2^. The CARG score was calculated retrospectively for each patient based on these variables.

Toxicities were described and graded according to the Common Terminology Criteria for Adverse Events version 5 (CTCE v5.0). In this system, AEs are categorized on a scale from grade 1 to grade 5 according to their severity based on a general guideline. Grade 1 corresponds to mild symptoms not requiring intervention; Grade 2 represents moderate symptoms that may limit instrumental activities of daily living; grade 3 corresponds to severe or medically significant symptoms that often require hospitalization or limit self‑care activities of daily living; grade 4 reflects life‑threatening consequences requiring urgent intervention, and grade 5 denotes death related to the AE [17]. The maximum grade for each type of toxicity was recorded for each patient.

Descriptive statistics were used to summarize patient, tumor and treatment characteristics. Statistical analyses were performed using scripts written in the Python programming language, employing the statistical functions available in the SciPy software. Spearman’s rank correlation coefficient was used to evaluate the relationship between the CARG score and the maximum grade of toxicity for each patient. A p-value < 0.05 was considered statistically significant.

## Results

A total of 283 patients were eligible for the present analysis. The median age was 73 years (range: 65-93 years), 56.2% (n = 159) were male and 65.4% (n = 185) had an ECOG PS of 0. The median BMI and SCr were 25.2 Kg/m^2^ and 0.86mg/dL, respectively. The median SHb was 12.4g/dL (range: 6.2-16.4g/dL).

Most of our population had excellent or good hearing (n = 258, 91.2%), could take their own medicine without help (n = 238, 84.1%), walk one block without limitation (n = 186, 65.7%) and did not have their social activities interfered by physical health or emotional problems (n = 177, 62.5%). Almost all patients did not report a fall in the past six months (n =281, 99.3%).

One hundred and sixty-one patients (56.9%) had gastrointestinal cancer, while 49 (17.3%) had genitourinary tract cancer; 34 (12%) had breast cancer; 24 (8.5%) had gynecological cancer, and 15 (5.3%) had head and neck cancer. More than half of patients had metastatic disease (n = 167, 59%) and were under a first-line systemic therapy (n = 217, 76.7%) with a standard dose (n = 216, 76.3%) polychemotherapy regimen (n = 156, 55.1%). The baseline characteristics of the population are described in Table 1.

**Table 1:** Patient Characteristics (N = 283) BMI: body mass index; GI: gastrointestinal; GU: genitourinary

Characteristic	
Age, years - median (range)	73 (65-93)
Sex – no. (%)	
Male	159 (56.2)
Female	124 (43.8)
BMI, Kg/m^2^ - median (range)	25.2 (14.3 – 41)
ECOG PS – no. (%)	
0	185 (65.4)
1	77 (27.2)
2	21 (7.4)
Serum creatinine, mg/dL - median (range)	0.86 (0.24 – 3.18)
Serum hemoglobin, g/dL - median (range)	12.4 (6.2 - 16.4)
Tumor type – no. (%)	
GI cancer	161 (56.9)
GU cancer	49 (17.3)
Breast cancer	34 (12)
Gynecological cancer	24 (8.5)
Head and neck cancer	15 (5.3)
Cancer stage	
Localized	116 (41)
Metastatic	167 (59)
Treatment dosage – no. (%)	
Standard	216 (76.3)
Reduced	67 (23.7)
Number of chemotherapy agents – no. (%)	
Monochemotherapy	127 (44.9)
Polychemotherapy	156 (55.1)
Chemotherapy line – no. (%)	
First line	217 (76.7)
Second line	48 (17)
Third line	16 (5.7)
Fourth line	2 (0.7)
How is your hearing?	
Excellent or good	258 (91.2)
Fair	22 (7.8)
Poor	3 (1.1)
Totally deaf	0
Number of falls in the past six months	
1 or more	2 (0.7)
None	281 (99.3)
Can you take your own medicines?	
Without help	238 (84.1)
With some help	45 (15.9)
Completely unable	0
Does your health limit you in walking one block?	
Limited a lot	18 (6.4)
Limited a little	79 (27.9)
Not limited at all	186 (65.7)
During the past four weeks, how much of time has your physical health or emotional problems interfered with your social activities?	
All of the time	1 (0.4)
Most of the time	10 (3.5)
Some of the time	54 (19.1)
A little of the time	41 (14.5)
None of the time	177 (62.5)

Overall, 238 (84.1%) patients experienced a chemotherapy-related toxicity event. Grade 2 toxicities were observed in 139 patients (49.1%), while grade 3-4 toxicities occurred in 93 patients (32.9%). Among patients aged 65-75 years, 30.2% (n = 54) had a grade 3-4 toxicity, compared to 36.3% (n = 33) in the 76-85 age group and 38.5% (n = 5) in the 86-95 age group.

The most common toxicities reported were fatigue (n = 88, 31.1%) and neutropenia (n = 80, 28.3%). 

**Figure 1:**
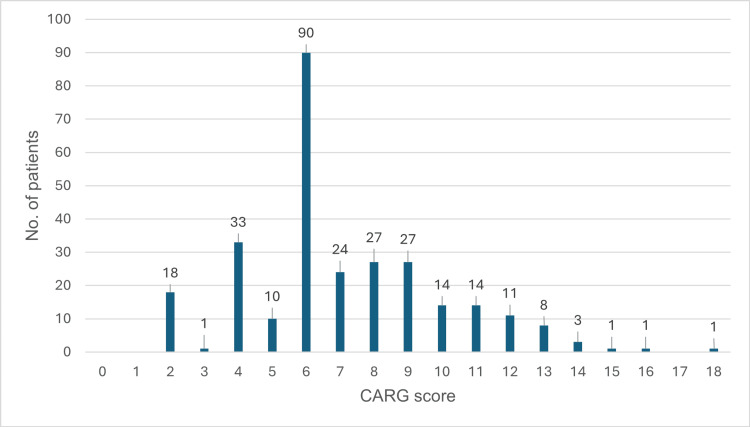
Distribution of the CARG score in the study population (N = 283) CARG: Cancer and Aging Research Group

The median CARG risk score was 6 (range: 2 - 18) and 62 (21.9%) patients were considered low risk for grade 3 to 5 toxicity, whereas 168 (59.4%) and 53 (18.7%) were considered intermediate and high risk, respectively. The numbers of patients with each scoring variable are shown in Figure 1.

The incidence of grade 3 to 4 chemotherapy-related adverse event based on the CARG risk scores was as follows: low (22.6%), intermediate (41.1%), and high (79.2%). The chemotherapy-related adverse events per CARG risk score are described in Table 2.

**Table 2:** Chemotherapy‑related adverse events per CARG risk group (N = 283) CARG: Cancer and Aging Research Group; CTCAE: Common Terminology Criteria for Adverse Events Version 5.0. “n” indicates the number of patients. All percentage values are rounded to the nearest whole number.

Toxicities per CTCAE v5.0	Low-risk group (62 patients)		Intermediate-risk group (168 patients)		High-risk group (53 patients)	
	All grades (n, %)	G3-4 (n, %)	All grades (n, %)	G3-4 (n, %)	All grades (n, %)	G3-4 (n, %)
Blood and lymphatic system disorders						
Anemia	2 (3)	0	6 (4)	3 (2)	9 (17)	7 (13)
Febrile neutropenia	0	0	1 (1)	1 (1)	0	0
Cardiac disorders						
Myocardial infarction	0	0	1 (1)	1 (1)	0	0
Eye disorders						
Watering eyes	0	0	1 (1)	0	0	0
Gastrointestinal disorders						
Constipation	0	0	3 (2)	0	0	0
Diarrhea	4 (6)	1 (2)	18 (11)	3 (2)	8 (15)	0
Dry mouth	2 (3)	0	1 (1)	0	0	0
Mucositis oral	2 (3)	1 (2)	8 (5)	2 (1)	1 (2)	0
Nausea	2 (3)	0	14 (8)	1 (1)	5 (9)	0
Vomiting	3 (5)	0	4 (2)	1 (1)	2 (4)	1 (2)
General disorders and administration site conditions						
Edema limbs	0	0	1 (1)	0	1 (2)	0
Fatigue	20 (32)	1 (2)	43 (26)	9 (5)	25 (47)	13 (25)
Investigations						
Alkaline phosphatase increased	0	0	0	0	1 (2)	0
Blood bilirubin increased	0	0	1 (1)	0	0	0
Creatinine increased	0	0	6 (4)	1 (1)	1 (2)	0
Neutrophil count decreased	15 (24)	9 (15)	48 (29)	31 (18)	17 (32)	11 (21)
Platelet count decreased	0	0	21 (13)	9 (5)	8 (15)	5 (9)
Anorexia	5 (8)	0	13 (8)	2 (1)	7 (13)	0
Hypokalemia	0	0	0	0	1 (2)	0
Hyponatremia	1 (2)	1 (2)	0	0	1 (2)	1 (2)
Nervous system disorders						
Dysgeusia	0	0	1 (1)	0	0	0
Ischemia cerebrovascular	1 (2)	0	0	0	0	0
Peripheral sensory neuropathy	8 (13)	0	17 (10)	1 (1)	5 (9)	1 (2)
Presyncope	0	0	0	0	1 (2)	0
Skin and subcutaneous tissue disorders						
Dry skin	0	0	1 (1)	0	0	0
Nail changes	1 (2)	0	2 (1)	1 (1)	0	0
Palmar-plantar erythrodysesthesia syndrome	5 (8)	1 (2)	7 (4)	2 (1)	6 (11)	3 (6)
Skin hyperpigmentation	0	0	1 (1)	0	0	0
Vascular disorders						
Hypotension	0	0	3 (2)	0	3 (6)	0
Peripheral ischemia	0	0	1 (1)	1 (1)	0	0
Thromboembolic event	0	0	0	0	1 (2)	0

A statistically significant correlation was observed between maximum toxicity grade and the CARG toxicity score (p = 2.8×10⁻⁶). Nonetheless, the strength of this correlation was limited (Spearman R = 0.27).

## Discussion

Older age is associated with a higher risk of developing treatment-related toxicity, and the etiology is multifactorial.

Aging is associated with redistribution of adipose tissue, loss of muscle mass, and reduction in the percentage of total body water, resulting in a decreased volume of distribution for many drugs. Additionally, older adults typically experience a decline in glomerular filtration rate, impairing the excretion of cytotoxic agents and their active metabolites. Notably, for every 10mL/min decrease in creatinine clearance, the risk of chemotherapy-related toxicity increases by approximately 12%. These physiological changes, combined with comorbidities, significantly alter the pharmacokinetics of the antineoplastic therapies [8,14].

Beyond physiological factors, nutritional status, psychological well-being, and polypharmacy also contribute to toxicity risk [11]. Approximately 13% of older adults with cancer have depression, and higher depressive symptom burden has been associated with an increased risk of severe chemotherapy-related toxicity [18].

Our study highlights the vulnerability of older cancer patients, with 84.1% experiencing at least one chemotherapy‑related AE, and the incidence increases progressively with advancing age. These findings came despite a seemingly healthy cohort: most patients reported being able to walk a block without limitation, manage their own medication without help, and maintain social functioning. Only two of 283 patients reported a fall within the previous six months, and 65.4% had an ECOG PS of 0. This reinforces that ECOG PS alone is a poor predictor of chemotherapy-related toxicity in older adults.

The median age in our cohort was 73 years, similar to that in the original study by Hurria et al. Evidence suggests that individuals in their seventh decade of life are particularly susceptible to treatment‑related toxicity, likely reflecting the cumulative physiological consequences of aging [1].

The incidence of grade 3-4 toxicity in our cohort was 32.9%, consistent with other real-world studies but substantially lower than the 58% reported by Hurria et al. [1,6,8]. Importantly, compared to the latter cohort, our population had fewer patients reporting recent falls (0.7% vs. 18%) and fewer receiving a polychemotherapy regimen (55.1% vs. 70%), both factors strongly associated with poor outcomes in older patients [1,19].

Anemia is another recognized risk factor for chemotherapy-related toxicity, as it increases susceptibility to myelosuppression and independently predicts hospitalization and mortality in the geriatric population [1]. Conversely, overweight and obesity have been reported as protective factors [2]. In our sample, 14.8% of patients had anemia (SHb < 10g/dL in women, and SHb < 11g/dL in men), comparable to that reported by Hurria et al. However, only 3.5% were underweight, and more than half (51.9%) were overweight or obese. This difference in nutritional profile may partly explain the lower incidence of grade 3-4 events observed.

Fatigue and neutropenia were the most common toxicities reported (31.1% and 28.3%, respectively), aligning with several other real-world cohorts. A study of 529 adults with advanced non-small cell lung carcinoma demonstrated a strong association between older age and higher fatigue burden, with patients older than 65 years having a 0.17 point higher mean fatigue grade than younger individuals. Neutropenia is similarly well known to be more frequent in older adults due to reduced bone marrow reserve and limited regenerative capacity [1,8,15,20,21].

Given the above, and the fact that many older adults are less willing to trade off quality of life for longer survival, the use of reliable prediction tools for treatment-related toxicity is essential. The CARG score has been validated internally and externally across diverse populations and tumor types for this matter [2,8,10,13-16].

Our study found a statistically significant correlation between the CARG score and the severity of chemotherapy-related toxicity in an older Portuguese population (p = 2.8×10⁻⁶). The incidence of grade 3-4 toxicity clearly increased across CARG risk categories (low: 22.6%, intermediate 41.2%, and high 79.2%), supporting its clinical utility.

Although the correlation was limited (Spearman R = 0.27), our cohort displays some characteristics that make it different from the original American population described by Hurria et al., including a higher proportion of men, more gastrointestinal tumors, fewer underweight patients, fewer receiving a polychemotherapy regimen, and potential cultural differences, which may partly explain this result.

To the best of our knowledge, this is the first study evaluating the correlation between the CARG risk score and chemotherapy toxicity in older Portuguese patients with solid tumors. Our relatively large data provides a unique insight into the real-world toxicity profile in older adults at a Portuguese Cancer Center.

However, the present study has some limitations, such as its retrospective design, which implies a risk of information bias due to variability in documentation, and its single‑center setting. Besides, we excluded patients treated with targeted therapies or immunotherapy, which are becoming increasingly used drugs, with a specific toxicity profile.

It is also important to note that the CARG score focuses primarily on grade 3 to 5 toxicities. Nevertheless, low-grade toxicities can be highly impactful in the elderly population. Kalsi et al. found that 35% of dose modifications and 39.1% of early chemotherapy discontinuations were due to low-grade toxicities [1,22]. In our study, 49.1% of patients experienced a grade 2 AE, with fatigue, neutropenia and diarrhea being the most common.

Future predictive models should incorporate the impact of low-grade AEs as well as toxicities associated with targeted and immunotherapy agents.

Regardless, our findings support the use of the CARG score as a complementary tool in Portuguese clinical practice to help anticipate toxicity and guide shared decision-making.

## Conclusions

Our study provides real-world evidence on the chemotherapy-toxicity profile of older Portuguese patients with solid tumors and demonstrated that the CARG score is significantly associated with treatment-related toxicity in this population. Nevertheless, the correlation was modest, underscoring the need to identify additional factors that contribute to a higher vulnerability to treatment-related AEs.

Future research should prioritize prospective, geriatric‑focused studies that incorporate modern treatments, such as targeted agents and immunotherapy, and that evaluate the impact of both high‑ and low‑grade toxicities. Ultimately, improving toxicity prediction is essential to individualize treatment strategies for this rapidly growing older patient population.
